# Model Evaluation of the Influence of the Plunger Stroke on Functional Parameters of the Low-Pressure Pulse Gas Solenoid Injector

**DOI:** 10.3390/s21010234

**Published:** 2021-01-01

**Authors:** Dariusz Szpica, Michał Kusznier

**Affiliations:** 1 Faculty of Mechanical Engineering, Bialystok University of Technology, 45C Wiejska Str., 15-351 Bialystok, Poland; 2 Doctoral School, Bialystok University of Technology, 45A Wiejska Str., 15-351 Bialystok, Poland; michal.kusznier.sd-1037@student.pb.edu.pl

**Keywords:** combustion engines, alternative fuel supply, gas solenoid injector, modeling

## Abstract

The article presents a model-based evaluation of the impact of the plunger stroke on functional parameters of the low-pressure pulse gas solenoid injector. A reduced-order physics-based mathematical model was used to achieve this goal. The model was built on the basis of specified simplifications of the process, considering the forces that cause the plunger to move and the forces constituting resistance to its displacement. The implementation of a mathematical description in to the Matlab-Simulink environment allowed one to determine the characteristic values of operation of the Valtek Rail Type-30 injector, including plunger displacement courses. Calculations made with the assumption of the factory plunger stroke confirmed the validity of the model. The differences in opening and closing times were below 3% in comparison to the values given in the objects technical information. By assuming a specific plunger stroke, the functional relationships of opening and closing times were determined. The results showed a distortion of the force–response dependence for different plunger strokes. Results presented in the article can be used to support control-oriented modeling of systems incorporating pulsed gas dosing devices, such as combustion engines or gas turbines. More specifically, the proposed method can be used to pre-calibrate the delay time of the injector operation.

## 1. Introduction

Despite the unstable situation of the oil market, interest in alternative fuels used in transport, in particular liquefied petroleum gas (LPG), continues to grow [1,2]. The prevalence of gaseous fuels is also supported by legal regulations, i.e., Corporate Average Fuel Economy (CAFÉ), Alternative Motor Fuels Act (AMFA) [3,4], which is why their shares are growing [5]. On the other hand, the ubiquitous downsizing of engines [6] raises new challenges for alternative power systems. This is compounded by modifications to the combustion process, such as Controlled Auto-Ignition (CAI)/Homogeneous Charge Compression Ignition (HCCI) [7,8], High Pressure Direct Injection (HPDI), or Reactivity Controlled Compression Ignition (RCCI) [9], which are partly met by LPG systems using petrol injectors [10]. Emissions legislation concerning the vehicle homologation process is also a problem [11,12], in which Worldwide harmonized Light vehicles Test Cycle (WLTC) and Real Driving Emissions test (RDE) driving cycles play a dominant role [13]. A separate issue is the approval regulations for non-road and working machinery engines [14,15], where further restrictions are expected in the near future. All legislative actions, except for the reduction of toxic emissions of exhaust components, are aimed at reducing CO_2_ emissions [16,17], making it increasingly attractive to use methane or petroleum-based gaseous fuels [18,19,20,21,22].

The analyzed low-pressure, pulse gas injector is typically used in a wide range of Compressed Natural Gas (CNG) and (LPG) systems, including the latest generation of such solutions, combining the features of liquid and volatile phase injection (i.e., AC LLC STAG 500.4 [23]). The increasing popularity of new or retrofitted gas engines in certain European countries [2], boosts the rapid incremental development of such systems [24,25].

The main challenges pertaining to the development of gas injectors are the necessity to provide high fuel injection rates (over 300 times more fuel by volume compared to petrol engines) [26], and non-repeatability in injected fuel value (especially while approaching ballistic operation regime) [27,28]. There are high hopes of using a piezoelectric injector drive instead of a standard electromagnetic one, which will be able to improve the force–response relationship of the injector, as well as enable proper operation at times less than 2.5 × 10^−3^ s [29,30].

Computational simulation can help resolve the above challenges with considerable advantages to the level of insight and development time. The approaches used for modeling (gas) injectors can be divided into several categories depending on the level of physics involved and methods incorporated:

- physical zero-, single-, or multidimensional [4,31,32];
- analytical [33,34,35];
- empirical [36,37,38];
- Finite Element Method (FEM) in the mechanical part [30,39,40];
- FEM in the electrical part [41,42];
- Computational Fluid Dynamics (CFD) in the flow part [43,44,45].

Thus, building a mathematical model of how the low pressure pulse gas solenoid injector works is a difficult task, but with some simplifying assumptions it is possible. A universal, simple mathematical model can be widely used in evaluating the functional parameters of an injector or testing new solutions (prototypes).

In such an approach, the mathematical model of the injector combines electrical, mechanical, and hydraulic sub-models. In the electrical part, the mathematical description focuses on the operation of the transistor key which controls the operation of the injector. This feature limits the current value when the injector is fully opened, which prevents it from heating [46], and is practically implemented as a Pulse-Width Modulation signal (PWM) signal [47,48]. The biggest problem in modeling the electrical part is determining the working parameters of the coil—mainly inductance. A large part of the mathematical model describing the operation of an electromagnetic circuit with a coil is based on an air coil without a movable core [49,50,51,52,53]. If it already tries to take into consideration the movable core, it is converted to an air coil in the end anyhow [54,55,56,57,58].

The movement of the injector plunger is the main aspect of analysis in the mechanical part [32,36,59]. A prime issue here is to estimate the friction parameters. The methods here involve the friction process [60] that can be applied and the aerodynamic drag force [61,62]. The hydraulic part is the calculation of the flow process through the injector [19,63]. The mathematical descriptions of hydraulic electro-valves can be successfully applied here [64].

Modeling the operation of the LPG vapor phase pulse injector specifically is not a common issue raised in scientific studies. The biggest problem boils down to determining the inductance of a coil with a moving core. The simplest method is to experimentally determine the inductance as a function of the displacement of a movable core [65]. Such an approach allows for differences in opening and closing times of the injector of 2.05% and 2.27% according to the manufacturer’s declaration. The dependency of inductance on the temporary value of voltage, resistance, and frequency of the excitation is presented, for instance, in the work of Borawski [38]. The verification of the whole model describing the operation of the gas injector was performed, by the mentioned authors, using the noncontact method. The Casio Exilim EX-FH100 fast camera was used for this purpose. The calculations were found to be consistent with the experimental studies, although one has to point out that the experimental matrix used for model validation was rather scarce.

Partial models of various phases of injector operation were also proposed [26,66]. Then, it allows for the possibility to describe, for instance, the course of current development or the disappearance of the nozzle flow. However, most of the partial models used in this approach are based on the characteristics obtained from experimental studies.

For that reason, it is necessary to develop a more complex and innovative mathematical model describing the operation of the LPG vapor phase pulse injector. Therefore, the authors propose an innovative approach in describing the inductance of the coil based on its geometric dimensions, material properties, and the momentary position of the movable core (plunger).

Bearing the above methodological development targets, the aim of the study was to assess the impact of the plunger stroke on the functional parameters of the pulse low-pressure gas injector. The opening and closing times were chosen as functional parameters, which determine the size of the fuel dose, and the time of its delivery. The obtained plunger displacement courses and the time shift of its occurrence in relation to the forced pulse are the basic information necessary in modeling the gas injection system. The mentioned characteristics can also be used for evaluating new gas fueling designs on the component and system level, as well as for retrofitting the engine to gas operation.

## 2. The Subject of the Modeling

**Figure 1:**
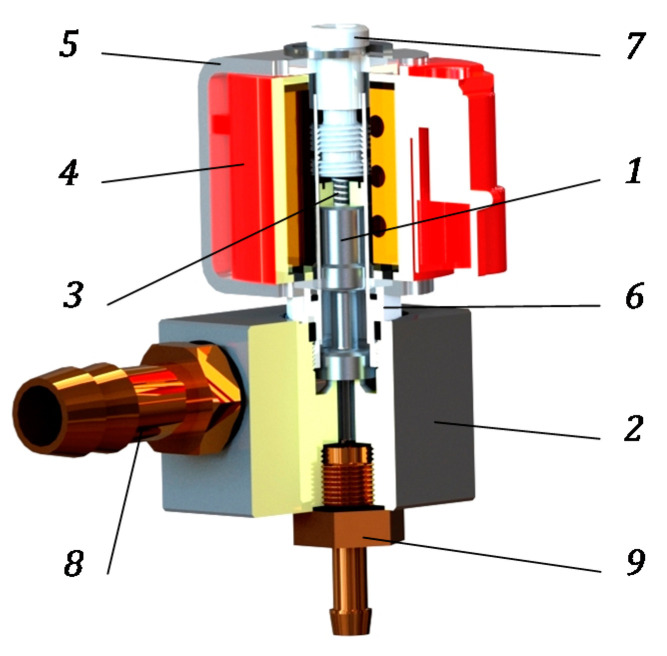
Valtek Rail Type-30 gas injector: *1*—plunger; *2*—corps; *3*—spring; *4*—coil; *5*—cramp; *6*—pilot; *7*—limiter; *8*—inlet nozzle; and *9*—outlet nozzle.

The subject of modeling was a Valtek Rail Type-30 plunger injector (Figure 1).

It is an injector with impulse action, where in the normal position plunger *1* is pressed to corps *2* with spring *3*. Without power supply, the flow valve is normally closed. When an electrical pulse appears on the coil *4* terminals, the solenoid circuit is closed using cramp *5*. Plunger *1* moves into pilot *6* as far as the resistance caused by the limiter *7*. When plunger *1* is raised, the gas flows from inlet nozzle *8* to outlet nozzle *9*. When the power supply fails, the coil *4* is moved to the closed position using spring *3*.

The flow rate of this type of injector is regulated by changing the opening time, outlet nozzle diameter, and plunger stroke. The last method is not recommended due to factory settings, but is feasible. As shown by the studies presented in [67], increasing the stroke increases the flow capacity. The technical data of the analyzed injector Valtek Rail Type-30 have been presented in Table 1.

## 3. Mathematical Modeling

**Figure 2:**
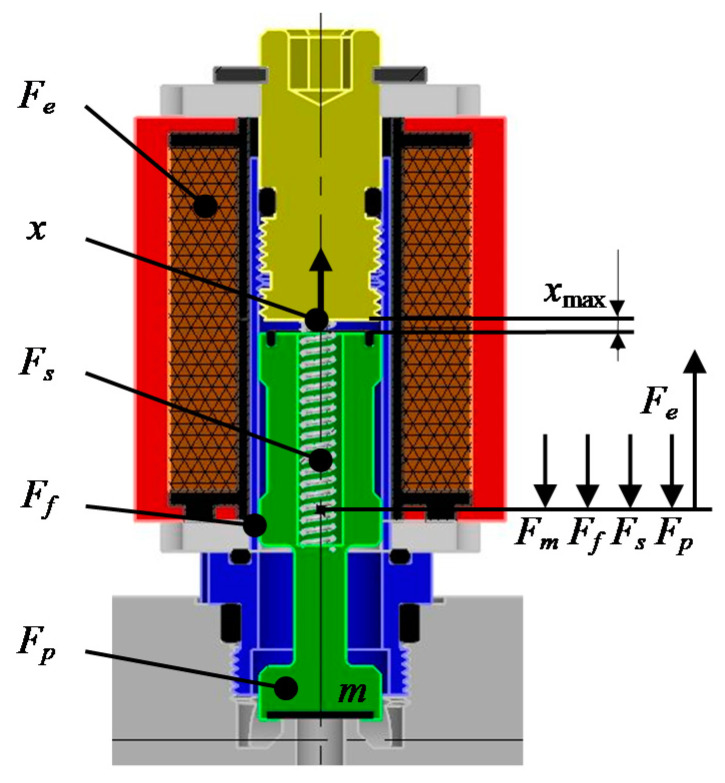
The scheme of the Valtek Rail Type-30 gas injector: *F_e_*—electromagnetic force; *F_f_*—frictional force; *F_s_*—spring force; *F_p_*—pressure force; *F_m_*—resistance force of mass inertia; *x*—plunger displacement.

The mathematical description of functioning of the gas injector was built on the basis of Figure 2. This is an upgrade of the mathematical model presented in [63] by the calculation part regarding the electromagnetic circuit in which the moving element is located inside the coil (modified description of the reluctance of the coil and, as a result, its inductance). In the paper [63], these parameters were defined by experimental results. 

Due to the high complexity of construction during the creation of the mathematical description, simplifying assumptions presented below were made:

- the plunger position depends on the resultant forces acting in the system, the effect of reflection from elements susceptible in the return positions is omitted;
- the electromagnetic force results from the operation of the coil without interference;
- the force generated by the pressure spring is due to its stiffness and pretension, the vibrations are omitted;
- the force from the pressure is distributed evenly and depends on the characteristic area and plunger position;
- the friction force responsible for damping takes into consideration different components depending on the position and movement of the plunger;
- the drag force due to its low impact on plunger movement was omitted.

The state of equilibrium of the plunger based on (Figure 2) can be described:

(1) $$F_{e}-F_{f}-F_{s}-F_{p}-F_{m}^{}=0$$

Each of the force components affecting the plunger position depends on many functional parameters (coefficients) as well as the position and movement of the plunger itself. The first presented in Equation (1) is the electromagnetic force forcing the plunger to move. This force is the result of an electromagnetic coil and its value can be written as (Equation (2)):

(2) $$F_{e}=\frac{1}{2}I^{2}\frac{dL\left(x\right)}{dx}$$

The value of the electromagnetic force *F_e_* is influenced by the current *I* supplying the coil and its inductance *L*. Due to the complexity of the entire electromagnetic circuit of Valtek Rail Type-30 injector and the ferromagnetic elements in its vicinity, the correct determination of the inductance is very difficult. An additional problem is to determine the changes of inductance as a function of plunger displacement or the frequency of electrical supply impulses. To calculate the current variation for the case of an electromagnetic circuit with a coil, the Faraday’s and Kirchhoff’s laws can be used (Equation (3)):

(3) $$\frac{dI}{dt}=\frac{1}{L\left(x\right)}\left(U-RI-\frac{dL\left(x\right)}{dx}\frac{dx}{dt}I\right)$$

Additionally, in this case (Equation (3)), the calculated current value depends on the inductance and its variation as a function of plunger displacement. As mentioned earlier in the literature descriptions [24,47,49,50,51,52,53,69,70], the actuator coil is modeled as a normal coil with an air gap. The attempts to change to a version with a moving component inside the coil [54,55,56,58] are also based on the relations that describe a normal coil with an air gap. 

**Figure 3:**
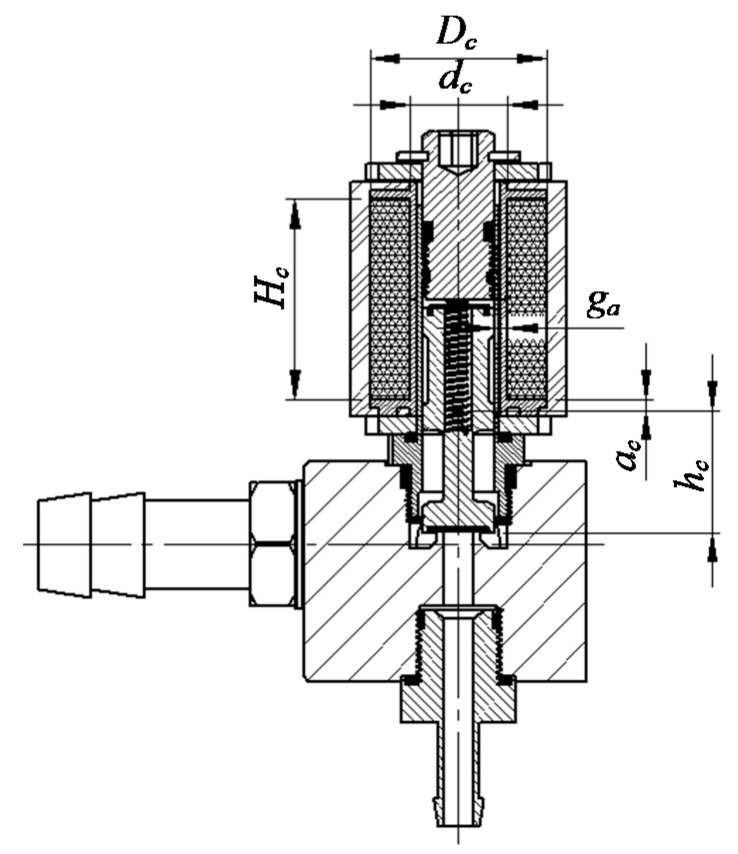
The generic scheme for calculating the inductance of the Valtek Rail Type-30 electromagnetic circuit: *a_c_*—distance of the plunger center of mass from the edge of the coil; *d_c_*—internal diameter of the coil; *D_c_*—outer diameter of the coil; *g_a_*—air gap between the plunger and the inner edge of the coil; *H_c_*—coil length; *h_c_*—length of plunger outside the valve cabinet.

They do not take into account the initial state of the plunger and even less the variability of inductance as a function of the plunger elevation, or the frequency of electrical supply pulses. The literature analysis concerning calculations of the inductance of an electromagnetic circuit with a moving core in the coil showed the lack of an unequivocal description considering the position of the core, properties of the materials used, or cramp asymmetry. Therefore, the article proposes an original approach. It consists of the fact that the commonly used (in literature) dependence describing the magnetic reluctance *R_M_* (Equation (4)) instead of operating with the distance from the core end (plunger) to the edge of the coil, the distance *a_c_*, which represents the distance from the center of mass to the edge of the coil, was proposed (Figure 3).

This is due to the fact that the plunger has an irregular shape and in its idle position is far from the coil edge. Additionally, Equation (4) considers the fact that there is an initial value of reluctance and as a result, inductance at the *x* = 0 m position, which was lacking in literature reports. The magnetic reluctance of the circuit according to Figure 3 can be calculated on the basis of a modified relation based on the previously presented literature dealing with the calculation of electromagnetic coils in the form of Equation (4).

(4) $$R_{M}=\frac{g_{a}}{\mu_{0}\pi xD_{m}}+\frac{g_{a}}{\mu_{0}\pi a_{c}D_{m}}=\frac{g_{a}}{\mu_{0}\pi a_{c}D_{m}}\left(1+\frac{a_{c}+x}{x}\right)$$

where the average coil diameter *D_m_* is defined as:

(5) $$D_{m}=\frac{D_{c}-d_{c}}{2}$$

Having the *R_M_* magnetic reluctance value and the number of *N* turns in the coil, inductance can be written using Equation (6).

(6) $$L\left(x\right)=\frac{N^{2}}{R_{M}}=\frac{\mu_{0}\pi a_{c}D_{m}N^{2}}{g_{a}}\left(1+\frac{x}{a_{c}+x}\right)$$

Taking as *L`:*

(7) $$L`=\frac{\mu_{0}\pi a_{c}D_{m}N^{2}}{g_{a}}$$

The final relation on the inductance can be written as Equation (8).

(8) $$L\left(x\right)=L`\left(1+\frac{x}{a_{c}+x}\right)$$

As a result, the inductance derivative was established as Equation (9).

(9) $$\frac{dL\left(x\right)}{dx}=\frac{a_{c}L`}{{\left(x+a_{c}\right)}^{2}}$$

In the case of a gas injector, frictional forces: static, kinetic, and viscous are responsible for damping the plunger movement. As mentioned in the simplifying assumptions, due to its small influence, the drag force was omitted. The general relationship to the friction force can be written as Equation (10):

(10) $$F_{f}=\left\{ \begin{array}{l} F_{fs}\;\;\;\;\;\;\;\;\;\mathrm{if}\;x=0,\;x=x_{\max}\\ F_{fk}+F_{fv}\;\mathrm{if}\;x\neq 0 \end{array}\right.$$

When the plunger is still, the force of static friction (Equation (11)) affects it:

(11) $$F_{fs}=\mu_{s}F_{N}$$

The pressure force *F_N_* can be taken at a level equivalent to that resulting from the plunger mass. This will represent a case where the injector will work in a horizontal position that can be applied in case of problems with the car body. The action of the solenoid should align the plunger with the pilot, which prevents its friction. However, with an asymmetrical cramp, the friction may intensify. The coefficient of friction is a characteristic of the mating surface. When the plunger moves, the static friction changes to the kinetic friction (Equation (12)) and the force of viscous friction (Equation (13)) occurs.

(12) $$F_{fk}=\mu_{k}F_{N}\mathrm{sgn}\left(x\right)$$

(13) $$F_{fv}=\mu_{v}\frac{dx}{dt}\mathrm{sgn}\left(x\right)$$

Both values of the friction forces *F_fk_* and *F_fv_* (Equations (12) and (13)) depend on the friction coefficients and are opposite to the direction of plunger movement. The resistance force of the plunger movement is also the spring force *F_s_* (Equation (14)). The direction of this force is constant and its value depends on the stiffness *k*, preload *x*_0_, and plunger displacement *x*.

(14) $$F_{s}=k\left(x_{0}+x\right)$$

In position *x* = 0 mm the plunger is pressed by the pressure force *F_g_* (Equation (15)). The value of this force depends on the surface area over *A*_1_ and under valve *A*_2_ (plunger) and the pressure value of gas *p*_1_ and inlet manifold *p*_2_. It is assumed that when the plunger is displaced by *x* > 1 × 10^−7^ mm, the pressure force fades away. The displacement tied to the pressure force fading away should be minimal and correlated with minimal steps of calculation.

(15) $$F_{g}=\left\{ \begin{array}{l} A_{1}p_{1}+A_{2}p_{2}\;\;\mathrm{if}\;x=0\\ 0\;\;\;\;\;\;\;\;\;\;\;\;\;\;\;\;\mathrm{if}\;x>1\times 10^{-7}\mathrm{mm} \end{array}\right.$$

In the analyzed case, the result of the calculation gives a plunger displacement. If the purpose of the calculation is flow parameters, the lumped element method can be used for this purpose [27], or the mathematical model presented in [64].

The last of the forces is the resistance force of mass inertia (Equation (16)). The value consists of the mass plunger *m* and its acceleration *a* = d^2^*x*/d*t*^2^. This force is contrary to the direction of plunger movement.

(16) $$F_{m}=m\frac{d^{2}x}{dt^{2}}$$

The plunger’s movement is limited by its turning points corresponding to *x* = 0 m and *x = x*_max_. Technically, this is called a plunger stroke and the return positions are settled in the valve seat and contact with the limiter. Depending on the calculation method, “hard” mechanical constraints may result in large acceleration gains and, as a result, temporary peaks in the resistance force of mass inertia. By putting the compounds in Equation (1), a system of first-order differential equations were obtained, Equation (17).

(17) $$\left\{ \begin{array}{l} \frac{dx}{dt}=v\\ \frac{dv}{dt}=\frac{F_{e}-F_{m}-F_{d}-F_{s}-F_{p}}{m}\\ \frac{dI}{dt}=\frac{1}{L\left(x\right)}\left(U-RI-\frac{dL\left(x\right)}{dx}\frac{dx}{dt}I\right) \end{array}\right.$$

**Figure 4:**
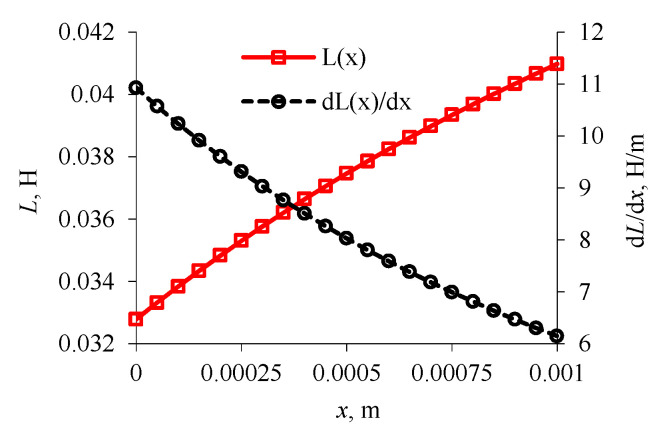
Calculated values of inductance and its derivative.

For solving the equation system (Equation (17)), Matlab-Simulink software was used. This software allows for easy implementation of empirical models, it is possible to introduce variability of boundary conditions or limitations of executive blocks. In the initial phase of calculation, values *L*(*x*) and d*L*/d*x* were determined from the relation of Equations (8) and (9). According to Figure 3, as input data, the following were assumed: *a_c_* = 3 × 10^−3^ m; *h_c_* = 13.86 × 10^−3^ m; *H_c_* = 23 × 10^−3^ m; *D_c_* = 20 × 10^−3^ m; *d_c_* = 11 × 10^−3^ m; *g_a_* = 1.4 × 10^−3^ m; *N_c_* = 500; μ_0_ = 4 × 10^−7^ H⋅m^−1^. On this basis, the value *L`* = 32.781 × 10^−3^ H and further values presented in Figure 4 were calculated.

Data related to the spring stiffness of the injector are taken from [65]. The characteristics of the spring were approximated by a linear function, from which the derivative was used to determine its stiffness equal to *k* = 832.83 N⋅mm^−1^ (*R*^2^ = 99.9%). To initiate the calculations, it was necessary to use the remaining input parameters and boundary conditions, which are presented in Table 2.

**Figure 5:**
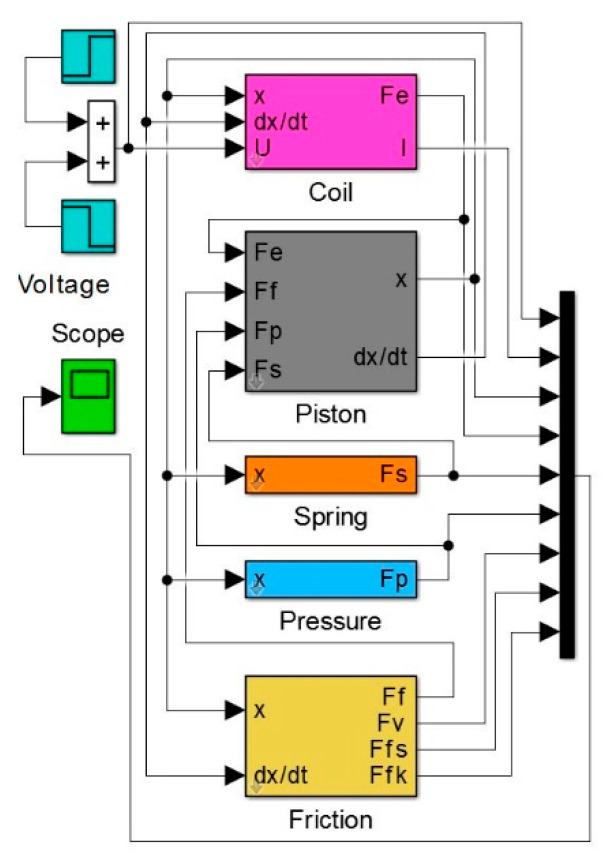
The block diagram in the Matlab-Simulink.

Presented as Equation (17), the system of differential equations was solved numerically with the implicit trapezoidal method combined with reverse differentiation (variable steps, min step 1 × 10^−7^ s). The maximum displacement of plunger *x*_max_ was taken as the control parameter in presented calculations. To achieve this goal, the limitations in the integration block of Simulink software were used, where each time an *x*_max_ value was set. The block diagram of the model implemented in the Matlab-Simulink environment is shown in Figure 5.

**Figure 6:**
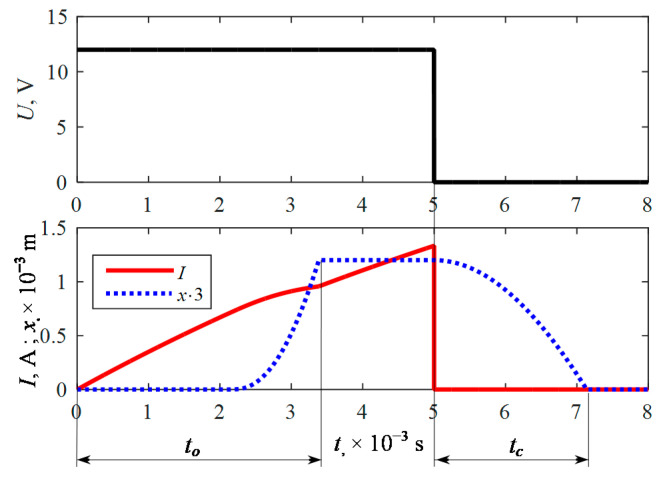
Variations of voltage, current in coil and plunger displacement were obtained by calculation at *x*_max_ = 0.4 × 10^−3^ m.

The first attempts were made to calibrate the model and to determine the correlation of the characteristic values obtained from the calculations with the manufacturer’s technical data. Basing on Equation (17) and the course of the pulse voltage *U*, it was possible to determine the course of current *I*, velocity, and as a result the plunger *x* displacement. Having the results of the calculations at *x*_max_ = 0.4 × 10^−3^ m (Figure 6) they were compared with the technical data of Valtek Rail Type-30 injector presented in Table 1.

**Figure 7:**
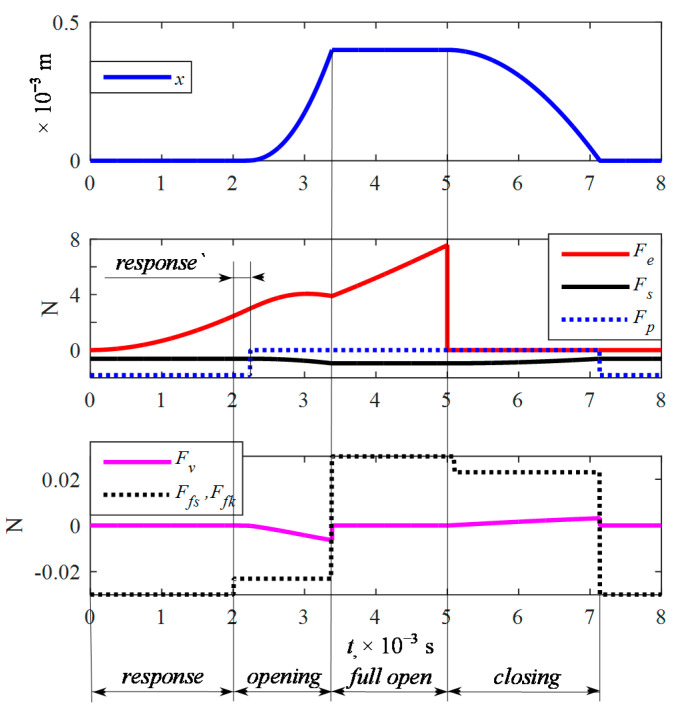
Variations of plunger displacement and acting forces were obtained by calculation at *x*_max_ = 0.4 × 10^−3^ m.

The opening time obtained from the calculations was *t_o_* = 3.386 × 10^−3^ s compared to 3.4 × 10^−3^ s declared by the manufacturer. The closing time obtained from the calculation was *t_c_* = 2.138 × 10^−3^ s compared to 2.2 × 10^−3^ s as declared by the manufacturer. This gives a comparison of 0.4% and 2.8% difference, which was considered sufficient to perform a comparative analysis by calculation. The calculations also allowed for the analysis of the operating forces (Figure 7). As can be observed, the electromagnetic coil force *F_e_*, the pressure force *F_p_*, and the spring force *F_s_* play a dominant role. Friction forces *F_fs_*, *F_fk_*, and *F_v_* affect the plunger movement in a small manner (more than 10 times lower value), which means a small damping.

## 4. Impact of Plunger Stroke on Functional Parameters of the Injector

**Figure 8:**
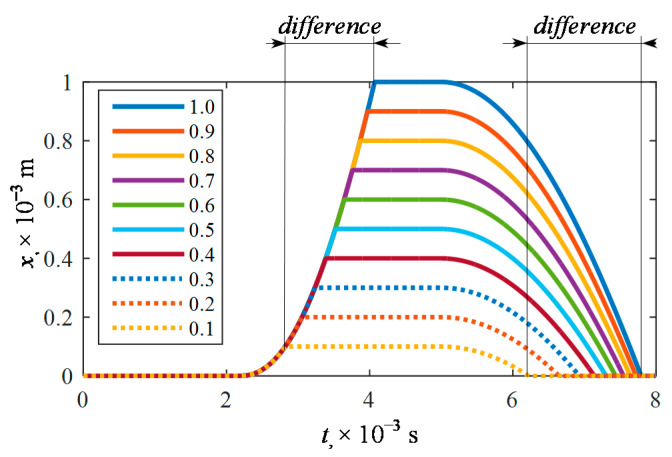
Calculated plunger displacement courses at different limit values *x*_max_ = (0.1…1.0) × 10^−3^ m.

By using the mathematical model of the gas injector implemented in the Matlab-Simulink environment, its maximum elevation was regulated in the range of *x*_max_ = (0.1…1.0) × 10^−3^ m. The plunger displacement courses were analyzed (Figure 8).

Differences in functional parameters amounting to more than 1 ms on the opening and closing side of the injector were noted (Table 3).

Additionally, it was noted that the opening time is on average two-times longer than the closing time. This is an unfavorable phenomenon as it significantly distorts the extortion–response relationship. With comparable opening and closing times, this relationship exists and the opening response time indicates a time shift in the response. In some gas Electronic Control Unit (ECU) solutions the injector selection option appears, which allows the opening response time to be corrected. Times of opening and closing are essential in the process of adaptation of the engine from original (petrol) to alternative gas [24,26] In the case of some engines, petrol injectors operate at short, 5 × 10^−3^ s and less opening pulses.

It is then required that the gas injector can also open during this time. If we have a Valtek Rail Type-30 injector, which has a very large opening response time and a total opening time of 3.4 × 10^−3^ s, it means that the minimum value of the injection time is *t_inj_* = 3.4 × 10^−3^ s with factory settings *x*_max_ = 0.4 × 10^−3^ mm. Therefore it is possible to try to reduce *x*_max_, which will allow to achieve a reduction of the minimum injection time, at which the injector will open. This must be confronted with an increase in outlet nozzle diameter or an increase in gas pressure. However, an increase in gas pressure, as shown in the calculation, has a significant effect on the opening time. The situation is even worse in the case of so-called “fuel injections”. When the engine load is rapidly increased, the control algorithm enriches the flammable mixture with extended injection time, or worse for the gas system, injects fuel several times, at very short times. It is found that short times that may be out of the range of full opening of the gas injector. This results in the engine ECU collecting information about the flammable mixture imbalance, resulting in a check engine.

**Figure 9:**
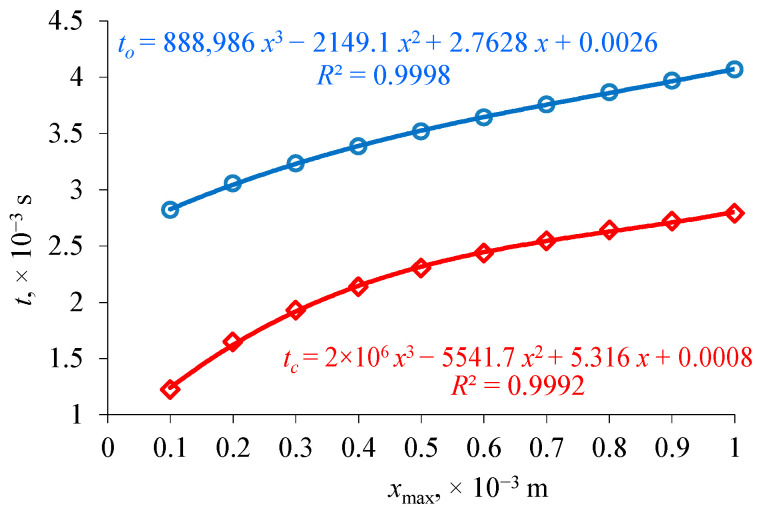
Relation of opening *t_o_* and closing time *t_c_* to plunger stroke *x*_max_.

The purpose of the study was to evaluate the impact of plunger *x*_max_ stroke on functional parameters limited to opening *t_o_* and closing time *t_c_*. As a result of the calculations performed, functional dependencies describing the variability of opening and closing times at different plunger strokes were determined. The characteristic values of the times were approximated by polynomials of the third degree (Figure 9) and obtained with determination factor R^2^ > 99.9%.

This confirmed the applicability of the mathematical model proposed in the study. Additionally, it was shown that the opening and closing times change by different values as the plunger stroke increases. This is an additional factor influencing the distortion of the force–response relation and consequently, the amount of fuel dose. During the use of the injector, its stroke may change under the influence of emerging contaminants (decrease) or as a result of wear of plunger damping elements (increase).

**Figure 10:**
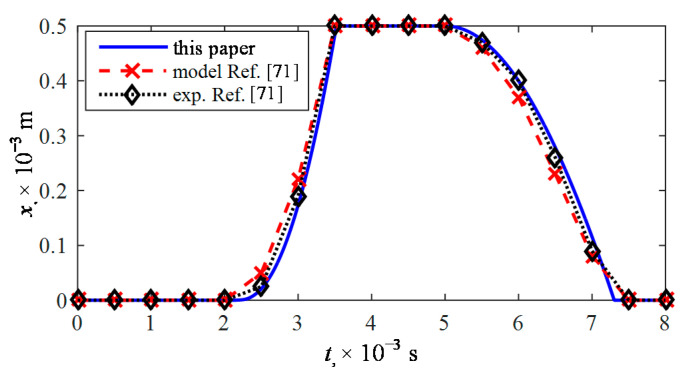
Calculation results verification.

In the final stage of the study, to verify the correctness of the mathematical description adopted in this paper and the way of obtaining the results, the course of the plunger lift with the value *x*_max_ = 0.5 × 10^−3^ m was compared with the results presented in [71]. The compliance of calculation courses mentioned in the paper [71] and the innovative model proposed in this paper (Figure 10) have been noted. At the same time, it was found that the presented model shows changes of plunger displacement more accurately and is more comparable to the results of experimental studies from the paper [71].

It was found that there were no calculation results in the published reports or experimental studies concerning the impact of plunger stroke on functional parameters of the injector. Therefore, in order to additionally verify the correctness of the applied mathematical model, the characteristic opening and closing times of the Valtek Rail Type-30 injector were compared to those available in only a few studies. The calculation and test results presented in Table 4 were compared each time to those declared by the manufacturer where the nominal stroke should be 0.5 × 10^−3^ m.

By comparing the results of calculations presented in this study with those available in the literature, we can see a more correct description of the electromagnetic drive which affects the process of opening the injector. Therefore, it is the mathematical model of a circuit with an electromagnetic coil that is the essence of this paper. The problem when referring to experimental results is the lack of confirmation from the authors of stroke control of the actuator before the measurement. This is essential because, as simulation studies have shown, this determines the opening and closing times. Only the method using high speed camera [72] can be considered as direct. However, the lack of confirmation of the plunger stroke value of the brand new injector and the fact that an additional element was attached to the plunger, which changes its mass, may result in a change in opening and closing times. Overall, using this method, there were differences of about (3 and 14%). In addition, the low number of measurement points, which has already been mentioned before, precludes detailed analysis. The measurement methods presented in [26,72] can be qualified as indirect, where as shown in Table 4, the differences in values are quite high, reaching 54%. 

The lack of possibility for direct comparison of model courses presented in this paper with the results of other authors resulted in planning our own future experimental tests regarding the impact of the plunger stroke on the functional parameters of the injector. Two options are being considered. Firstly, the use of a non-contact optical sensor, which requires interference with the injector body (cutting out its parts), which may change the inductance of the electromagnetic circuit to some degree, but will not load the plunger. The second variant is to use an inductive sensor and a needle fixed to the plunger. In this case, it is also expected that the circuit inductance and additionally the weight of the plunger will change. Only then will it be possible to confirm the correctness of the mathematical model presented in this paper.

## 5. Summary and Conclusions

The analysis presented in the paper aimed at estimating the opening and closing time of a variable stroke low-pressure gas injector. For this purpose, an in-house developed physics-based, control-oriented injector model was implemented in a Matlab-Simulink simulation. The conclusions are as follows:

1. The presented new mathematical model was successfully validated. The simulated opening and closing times differed respectively, by 0.4% and 2.8% from the declaration in the injector technical documentation.
2. The proposed mathematical description of the reluctance of the coil and its inductance are the basis of the modular model architecture. Further modifications can be introduced to this submodel, for improved simulation accuracy, by accounting for the reluctance of the electromagnetic circuit components and their location.
3. The plunger forces’ analysis showed the dominant role of electromagnetic coil force, pressure, and the spring force. On the other hand, the forces responsible for frictional damping are of an order of magnitude lower.
4. As the plunger stroke increases, the asymmetry of the opening time/closing time direct influence is noticeable, which may affect the amount of fuel dosing.
5. A comparison of the results with the calculations of other authors has shown compatibility. The proposed model is more responsive to the variability of forces and movement conditions.
6. The innovative concept of coil inductance modeling, incorporating the core dynamics, proposed in this paper is correct, was found to be valid within the constraints of the adopted literature reference data.

The obtained functional relations for opening and closing times as the plunger stroke increases can be used in the mathematical modeling of the operation of the internal combustion engine or during the calibration of the gas supply system under real conditions. The presented mathematical model of the gas injector can be successfully used to assess the influence of factors other than the presented ones, such as supply voltage, system pressure, or plunger mass on functional parameters. In the next research stage, the new model will be validated against real-world injector operational data.

## Figures and Tables

**Table 1:** The technical data of the Valtek Rail Type-30 injector. Data taken from Ref. [68].

Parameter	Value
nozzle size	4 × 10^−3^ m
piston stroke	0.4 × 10^−3^ m
coil resistance	3 Ω
opening time	3.4 × 10^−3^ s
closing time	2.2 × 10^−3^ s
maximum working pressure	4.5 × 10^3^ Pa
operating temperature	(−20… + 120) + 273.15 K

**Table 2:** The parameters and function conditions needed to initiate the simulation.

Parameter	Value
injection time	*t_inj_* = 5 × 10^−3^ s
mass of the piston and needle	*m* = 5 × 10^−3^ kg
resistance/impedance	*R* = 3 Ω
initial tension the spring	*x*_0_ = 0.75 × 10^−3^ m
coefficient of static friction	*μ_s_* = 0.61
coefficient of kinematic friction	*μ_k_* = 0.47
coefficient of viscous friction	*μ_v_* = 0.009 N⋅s⋅m^−1^
normal force	*F_N_* = *m g*
cross area over the valve	*A*_1_ = 32.56 × 10^−6^ m^2^
cross area under the valve	*A*_2_ = 12.56 × 10^−6^ m^2^
gas pressure	*p*_1_ = 1 × 10^5^ Pa + *p*_2_
inlet manifold pressure	*p*_2_ = 1 × 10^5^ Pa
**Boundary Conditions**	
at *t* = 0	*U* = 12 V; *x* = 0 m
after the time *t* = *t_inj_*,	*U* = 0 V

**Table 3:** The calculated values of opening *t_o_* and closing time *t_c_* at different maximum heights (stroke) plunger.

*x*_max_, × 10^−3^ m	0.1	0.2	0.3	0.4	0.5	0.6	0.7	0.8	0.9	1.0
***t_o_*, × 10^−3^ s**	2.82	3.05	3.23	3.39	3.52	3.64	3.76	3.87	3.97	4.07
***t_c_*, × 10^−3^ s**	1.22	1.65	1.93	2.14	2.30	2.43	2.55	2.64	2.72	2.79

**Table 4:** Comparison of Valtek Rail Type-30 injector opening and closing times.

Source	Method	*t_o_*, × 10^−3^ s	*t_c_*, × 10^−3^ s	Δ*t_o_*, %	Δ*t_c_*, %
Ref. [68]	manufacturer	3.40	2.20	−	−
this paper	modeling	3.386	2.138	−0.41	−2.82
Ref. [65]	modeling	3.47	2.15	2.05	−2.27
Ref. [71]	modeling	3.50	2.50	−2.94	13.64
Ref. [26]	experiment (current line)	3.30	−	−2.94	−
Ref. [71]	experiment (high speed camera)	3.50	2.50	2.94	13.64
Ref. [72]	experiment (1 pressure sensor)	3.10	2.54	−8.82	15.45
Ref. [26]	experiment (2 pressure sensor)	−	1	−	−54.55
Ref. [72]	experiment (vibration sensor)	2.70	2.45	−20.59	11.36

## Abbreviations and Acronyms

The following abbreviations and acronyms are used in this manuscript:

EFA	Example of First Abbreviation
AMFA	Alternative Motor Fuels Act
CAFÉ	Corporate Average Fuel Economy
CAI	Controlled Auto-Ignition
CFD	Computational Fluid Dynamics
CNG	Compressed Natural Gas
CO_2_	Carbon dioxide
ECU	Electronic Control Unit
FEM	Finite Element Method
HCCI	Homogeneous Charge Compression Ignition
HPDI	High Pressure Direct Injection
LPG	Liquefied Petroleum Gas
PWM	Pulse-Width Modulation signal
RCCI	Reactivity Controlled Compression Ignition
RDE	Real Driving Emissions test
WLTC	Worldwide harmonized Light vehicles Test Cycle
*A*_1_	cross area over the plunger, m^2^
*A*_2_	cross area under the plunger, m^2^
*a_c_*	distance of the plunger center of mass from the edge of the coil, m
*d_c_*	internal diameter of the coil, m
*D_c_*	outer diameter of the coil, m
*F_e_*	electromagnetic force, N
*F_f_*	friction force, N
*F_fk_*	kinetic friction force, N
*F_fs_*	static friction force, N
*F_fv_*	viscous friction force, N
*F_m_*	resistance force of mass inertial, N
*F_N_*	normal force, N
*F_p_*	pressure force, N
*F_s_*	spring force, N
*g*	gravitational acceleration, m⋅s^−2^
*g_a_*	air gap between the plunger and the inner edge of the coil, m
*H_c_*	coil length, m
*h_c_*	length of plunger outside the valve cabinet, m
*I*	current, A
*k*	spring stiffness, N⋅m^−1^
*L*	inductance, H
*m*	plunger mass, kg
*N_c_*	number of turns of coil, -
*p*_1_	gas pressure, Pa
*p*_2_	inlet manifold pressure, Pa
*R*	resistance, Ω
*R_M_*	magnetic reluctance, H^−1^
*t_c_*	closing time, s
*t_inj_*	injection time, s
*t_o_*	opening time, s
*U*	voltage, V
*x*	plunger displacement, m
*x*_0_	initial tension the spring, m
Δ*t_o_*	opening time difference, %
Δ*t_c_*	closing time difference, %
μ_0_	permeability of vacuum, H⋅m^−1^
μ_k_	coefficient of coefficient of kinetic friction, -
μ_s_	coefficient of static friction, -
μ_v_	coefficient of coefficient of viscous friction, N⋅s⋅m^−1^
